# Erythropoietin reduces experimental autoimmune encephalomyelitis severity via neuroprotective mechanisms

**DOI:** 10.1186/s12974-017-0976-5

**Published:** 2017-10-13

**Authors:** M. Moransard, M. Bednar, K. Frei, M. Gassmann, O. O. Ogunshola

**Affiliations:** 1 Department of Internal Medicine, Section of Clinical Immunology, University Hospital Zürich, Zurich, Switzerland; 2 Institute of Veterinary Physiology and Zurich Center of Integrative Human Physiology (ZIHP), Vetsuisse Faculty, University of Zurich, Winterthurerstrasse 260, CH-8057 Zurich, Switzerland; 3 Department of Neurosurgery, University Hospital Zurich, CH-8006 Zurich, Switzerland; 4 Universidad Peruana Cayetano Heredia (UPCH), Lima, Peru

**Keywords:** Multiple sclerosis, Myelin, Immunomodulation, Epo, EAE, Experimental autoimmune encephalomyelitis, Neuroprotection

## Abstract

**Background:**

Treatment with erythropoietin (Epo) in experimental autoimmune encephalomyelitis (EAE), the rodent model of multiple sclerosis (MS), has consistently been shown to ameliorate disease progression and improve overall outcome. The effect has been attributed to modulation of the immune response and/or preservation of the central nervous system (CNS) tissue integrity. It remains unclear, however, if (a) Epo acts primarily in the CNS or the periphery and if (b) Epo’s beneficial effect in EAE is mainly due to maintaining CNS tissue integrity or to modulation of the immune response. If Epo acts primarily by modulating the immune system, where is this modulation required? In the periphery, the CNS or both?

**Methods:**

To address these questions, we used two well-characterized transgenic mouse strains that constitutively overexpress recombinant human Epo (rhEpo) either systemically (tg6) or in CNS only (tg21) in a MOG-induced EAE model. We assessed clinical severity, disease progression, immunomodulation, and CNS tissue integrity, including neuronal survival.

**Results:**

Although disease onset remained unaffected, EAE progression was alleviated in transgenic animals compared to controls with both lines performing equally well showing that expression of Epo in the periphery is not required; Epo expression in the CNS is sufficient. Immunomodulation was observed in both strains but surprisingly the profile of modulation differed substantially between strains. Modulation in the tg21 strain was limited to a reduction in macrophages in the CNS, with no peripheral immunomodulatory effects observed. In contrast, in the tg6 strain, macrophages were upregulated in the CNS, and, in the periphery of this strain, T cells and macrophages were downregulated. The lack of a consistent immunomodulatory profile across both transgenic species suggests that immunomodulation by Epo is unlikely to be the primary mechanism driving amelioration of EAE. Finally, CNS tissue integrity was affected in all strains. Although myelin appeared equally damaged in all strains, neuronal survival was significantly improved in the spinal cord of tg21 mice, indicating that Epo may ameliorate EAE predominantly by protecting neurons.

**Conclusions:**

Our data suggests that moderate elevated brain Epo levels provide clinically significant neuroprotection in EAE without modulation of the immune response making a significant contribution.

**Electronic supplementary material:**

The online version of this article (10.1186/s12974-017-0976-5) contains supplementary material, which is available to authorized users.

## Background

Multiple sclerosis (MS) results in long-term disability and reduced life expectancy. A chronic autoimmune disease of the CNS, MS is classically characterized by CNS inflammation, widespread demyelination, and damage of oligodendrocytes and neurons. Substantial irreversible axonal damage and neuronal cell death are thought to be an essential cause of non-remitting clinical disability in primary and secondary progressive MS. So far, no clinically effective therapy has been established to prevent axonal and neuronal damage in this neuroinflammatory disease, and despite much research, understanding of the etiology and pathogenesis of this condition remains limited.

Erythropoietin (Epo) is a hematopoietic growth factor that circulates in plasma and primarily controls the oxygen-carrying capacity of the blood [1]. Epo has an established safety profile for clinical use and has been extensively employed to increase red blood cell count during treatment of anemia [2] thereby avoiding the requirement for blood transfusions in dialysis and cancer patients [3, 4]. Originally believed to play a role restricted to stimulation of early erythroid precursor proliferation and differentiation, in recent years Epo has evolved into a promising candidate for neuroprotective approaches to diverse brain diseases [5–8], such as epilepsy [9], Parkinson’s disease [10], Alzheimer’s disease [11, 12], traumatic brain injury [13], ischemia [14–16], and diabetic neuropathy [17, 18]. Epo can cross the blood-brain barrier and is secreted within the CNS by astrocytes [19–21]. Under normal conditions, the Epo receptor (EpoR) is mainly expressed by neurons [22] but during insults such as hypoxia, ischemia, and trauma all cells including neurons, microglia, and astrocytes [19, 23, 24] are able to upregulate the Epo signaling cascade eliciting both autocrine and paracrine effects [18]. Although the exact mechanisms behind Epo’s neuroprotective effects are not well understood, its putative anti-inflammatory actions and enhancement of survival signals probably play a key role [7, 25–29].

To date, a substantial number of publications have shown that Epo and Epo derivatives have significant beneficial potential in various rodent models of MS [25, 27, 30–38]. Despite using significantly different treatment protocols, remarkably and indeed encouragingly, all the studies recorded a significant reduction in disease severity.

As treatment of MS will inevitably need to be long term, a significant limitation of most of the above studies was the relatively short duration of Epo treatment, varying from 7 to 14 days only, to circumvent detrimentally high increases in hematocrit. In this respect, the recent studies with Epo derivatives such as ARA290 [38], JM-4 [27], and Epobis [39] are encouraging, showing that, at least to some extent, the immunomodulatory effects of Epo can be separated from its erythropoietic properties in animal models of MS.

Taken together, these animal studies indicate that treatment of Epo and/or derivatives of Epo may be beneficial in MS, and indeed the first trials in humans were encouraging. The first proof-of-principle exploratory open label study (phase I/IIa) of recombinant human Epo (rhEPO) treatment in patients with chronic progressive MS, showed a significant improvement in patient clinical and electrophysiological motor functions that persisted for 3 to 6 months after cessation of Epo application. Importantly, the treatment protocols used had no adverse effects but blood-lettings to reduce hematocrit were required [40]. More recently, another randomized double-blind pilot study combined Epo with methylprednisolone in severe motor relapsing-remitting MS patients. Improvement in maximal distance walking was observed in the Epo group after 2 months and MRI data analysis showed a significant reduction in the number of T2WI lesions [41]. Despite these encouraging results, in the recent large-scale phase 2 trial of Epo in 52 patients with primary or secondary progressive MS, no beneficial effects of high-dose Epo treatment were detected [42]. No difference in the primary outcome, a composite measure of maximum gait distance, hand dexterity, and cognition from baseline to 24 weeks, between the EPO and the placebo group of the intention-to-treat populations, was observed and none of the secondary outcomes, neither clinical nor magnetic resonance imaging (MRI) measures, showed any significant differences. Although this study provides class 2 evidence that treatment with high-dose EPO is not an effective treatment in patients with progressive MS, the question if Epo treatment is effective in relapsing-remitting MS (RR-MS) patients remains open. Especially because the immunomodulatory properties of Epo are more likely to have an effect in the immune response-driven RR-MS stage rather than in the more immune quiescence progressive MS stages.

In short, to date, most studies indicate beneficial effects of Epo treatment in MS and EAE. However, there is very little consensus on its precise mechanism of action. Reported effects on the immune system are inconsistent between the different studies and direct neuroprotective effects of Epo in MS or EAE have not been convincingly established either.

Clearly better mechanistic insight will potentially allow (1) a more “mechanism of action”-focused approach to treatment of MS patients with Epo or its derivatives and (2) beneficial effects to be dissociated from possible negative side effects of Epo treatment, such as sustained elevated hematocrit levels, tumor growth, or prolonged suppression of inflammatory responses.

We performed a detailed study to analyze the pathological elements of EAE while distinguishing between Epo effects on the CNS directly versus peripheral effects influencing the immune response. We used two well-characterized transgenic mouse strains, called tg6 and tg21, that constitutively overexpress human Epo systemically (CNS and periphery, tg6) or in CNS only (tg21) [43–45]. We studied whether Epo acts directly on cells of the CNS or has a more systemic role in modulation of the autoimmune response in EAE. We assessed whether peripheral effects of Epo, such as increased erythropoiesis and modulation of the immune response, influence disease outcome.

Our data shows that Epo’s primary beneficial effect in EAE resides in the CNS, most likely by protecting neurons during EAE progression, and that immune modulation by Epo does not contribute significantly to amelioration of EAE.

## Methods

### Animals

All experiments were performed in accordance with Swiss animal protection laws and Zürich University institutional guidelines. The transgenic mouse line was generated by pronuclear injection of the full length human Epo cDNA driven by the human platelet-derived growth factor (PDGF) B-chain promoter and has been previously described [46, 47]. Two mice lines were generated; TgN(PDGFBEPO)321ZbZ (termed tg6) displayed 10–12-fold increased plasma and brain levels of Epo leading to hematocrit values of up to 0.9. The line was bred by mating hemizygous males to wild type (wt) C57BL/6 females and half the offspring were hemizygous for the transgene and half were wt thus serving as controls. The other transgenic line TgN(PDGFBEPO)322ZbZ (termed Tg21), bred to homozygosity, showed a fourfold increase of Epo levels in brain but not in blood plasma [43, 48]. Transgenic homozygous mice were backcrossed with C57Bl/6 mice for more than six generations to obtain wt littermates. In this study, male and female mice were used at 8–12 weeks of age.

### Antibodies

For flow cytometry, the following antibodies were used: CD3 (biotin), CD4 (PE), CD8 (FITC), CD11b (biotin), B220 (biotin) and Gr1 (FITC), which were obtained from BD PharMingen and F4/80 (FITC) from Serotec. CD11b (unlabeled), CD16/32 (Fc block), and CD45 (APC) were purchased from Biolegend and CD11c (clone N418, PE) from Caltag. The following antibodies were used for Western blotting and immunohistochemistry: anti-MBP, anti-smi35, anti-smi32 (1:1000, Sternen monoclonals), anti-neurofilament (Zymed, San Francisco, USA), anti-GFAP and anti-β-actin (Sigma). Secondary antibodies used were anti-rabbit HRP (Pierce), anti-rat Alexa 488 and anti-rabbit Alexa 594 (Molecular Probes/Invitrogen) and Streptavidin-APC-Cy7 (Biolegend).

### Induction of EAE

Mice were injected with 100 μg MOG35–55 (Anawa, Switzerland) in 200 μl PBS/CFA (DIFCO, USA) (1:1) subcutaneously on the right flank and with 300 ng Pertussis toxin (List Biological Laboratories, USA) intraperitoneal (i.p.) on day 0, followed by a boost of 300 ng Pertussis toxin at day 2 and 100 μg MOG35–55 in CFA into the left flank on day 7. The scoring of clinical symptoms was performed daily from day 7 onwards as described previously [49–51]. In all figures, preclinical, peak, early, and late (remission) refer to days 7, 17 ± 2, 24, and 30 ± 3 days post-immunization (p.i.) respectively.

### Cytokine multiplex

Spinal cord lysates were prepared using the Bio-Plex Pro™ Mouse Cytokine 23-plex Assay from BioRad according to the manufacturer’s instructions. Briefly, isolated spinal cords were washed with cell wash buffer, transferred to a 2-ml tissue grinder containing 500 μl of lysing solution and homogenized on ice. After a freeze cycle at − 70 °C, sonification on ice and centrifugation at 4500×*g* for 4 min, the supernatant was collected and the protein concentration determined. All samples were diluted to a final concentration of 0.5 mg/ml and stored at − 20 °C. Samples were analyzed on a Bio-Plex 200 system.

### Isolation of CNS mononuclear cells, lymphocytes, and splenocytes

CNS mononuclear cells were isolated as described previously [50]. Briefly, brains and spinal cords of animals perfused with Hanks’ balanced salt solution (HBSS) were minced with a scalpel blade and digested for 30′ at 37 °C in HBSS containing 50 mg/ml DNase I and 100 mg/ml Collagenase/Dispase (Roche). The digestion was quenched on ice and passed through a 100-μm nylon mesh (BD Biosciences) and centrifuged after which the pellet was resuspended in 30% Percoll (Sigma). The gradient was centrifuged at 29,000×*g* for 30 min at 4 °C (Kontron Instruments, Germany). The top layer containing myelin was removed by aspiration, and the interphase containing mononuclear cells was collected, diluted threefold with HBSS, collected by centrifugation at 300×*g* and resuspended in an appropriate volume of FACS buffer (2% FCS, 5 mM EDTA, 0.01% NaN_3_) in phosphate buffered saline (PBS). To isolate lymphocytes, inguinal lymph nodes were mashed through a 100-μm cell strainer, washed with HBBS and resuspended in FACS buffer. To obtain splenocytes, spleens were mashed through a 100-μm cell strainer followed by lysis of erythrocytes in ACK buffer (155 mM NH_4_Cl, 10 mM KHCO_3_, 0.1 mM EDTA, pH 7.2), several washes with HBBS and resuspension in FACS buffer.

### Flow cytometric analysis

Flow cytometric analysis was performed as described previously [50, 51]. Prior to staining with the appropriate antibodies, Fc receptors were blocked by incubation with anti-mouse CD16/32 (Fc block). 7-Amino-actinomycin D (7-AAD) was used to exclude non-viable cells. All flow cytometric data was obtained with a CyFlow flow cytometer (Partec, Germany) and data analysis was performed with FlowJo software. Infiltrating mononuclear cells were distinguished from CNS-resident microglia (CD45^int^/CD11b^+^/F4/80^+^) based on high CD45 expression. Within this population, the following cell types were distinguished using gating as indicated between brackets: CD4 T cells (CD3^+^/CD4^+^), CD8 T cells (CD3^+^/CD8^+^), T regs (regulatory T cells; CD4^+^/CD25^+^/FoxP3^+^), DCs (dendritic cells; CD11c^+^/B220^−^/Gr1^−^), pDCs (plasmacytoid DCs; CD11c^+^/B220^+^/Gr1^+^), B cells (CD11c^−^/B220^+^/Gr1^−^), macrophages (CD11c^−^/CD11b^+^/F4/80^+^).

### Quantitative polymerase chain reaction (qPCR)

Total RNA from cultured cells was extracted using the NucleoSpin-RNA II kit (Macherey-Nagel, Switzerland). RNA from mouse tissues was extracted by homogenization in TRIzol (Invitrogen) according to the manufacturer’s instructions. RNA was reverse-transcribed using random hexamers and AMV reverse transcriptase (Promega). The cDNA equivalent to 50 ng of total RNA was PCR-amplified in an ABI PRISM 7700 detection system using TaqMan Universal PCR Master Mix (PE-Applied Biosystems) and quantified using the 2^−ΔΔCT^ method using 18s rRNA as a housekeeping gene. Relative RNA levels are expressed as x-fold variations compared to control. Primers and probes for Taqman analysis for IL-1β, IL-2, IL-6, IL-10, IL-17, IL-23, TNFα, IFNγ, and TGFβ1 were purchased from Applied Biosystems.

### Epo radioimmuneassay (RIA)

Quantitation of Epo was performed on blood plasma as well as brain and spinal cord tissue lysate samples using EPO-Trac™ ^125^I RIA Kit according to manufacturer’s instructions (Diasorin, USA). Bound tracer was measured using a gamma counter (Gamma counter 670, Kontron, Germany).

### Western blotting

Experiments were carried out on brain lysates from transgenic and littermate controls. Tissue was homogenized in lysis buffer using a sterile dounce homogenizer. Samples were then centrifuged at 13,000 rpm at 4 °C for 10 min, supernatant extracted and stored at − 20 °C. Fifty micrograms of protein was run on a 10% SDS PAGE, transferred to a nitrocellulose membrane, blocked in 5% non-fat milk, and then incubated with primary antibodies overnight at 4 °C or 2 h at room temperature. After washing and incubation for 1 h with a horseradish peroxidase-conjugated secondary antibody, chemiluminescent detection was carried out. Normalization was achieved by reprobing filters with β-actin. Blot quantification was performed using ImageJ software (NIH, Bethesda, USA).

### Immunohistochemistry and histology

Mouse spinal cords were dissected and immediately frozen in liquid nitrogen. Using a cryostat, 10-μM sections were cut, fixed with 4% paraformaldehyde in PBS (pH 7.4) for 5 min at room temperature, then permeabilized in 0.1% Triton X-100 for 2 min and blocked with 4% normal goat serum. Sections were then incubated with primary antibodies overnight at 4 °C. Secondary antibodies were then applied for 2 h at room temperature followed by counterstaining with DAPI. To determine axonal damage and loss, cryostat serial sections from thoracic and lumbar spinal cord were stained for neurofilament, SMI32, and SMI35. Images were captured using a Zeiss Axiovert 200 M fluorescence microscope with an attached CCD camera. Five images were captured from individual brain sections per animal. Images were processed and quantified using NIH ImageJ software. After manual selection of the area of interest, the image was inverted and converted to binary and the number of axons analyzed using the particle count option. To assess the degree of demyelination, spinal cords were removed, fixed in 4% buffered formalin, and embedded in paraffin before staining with hematoxylin eosin (H&E) and luxol fast blue (LFB) as described recently [51]. At least four animals per group and four images per animal were analyzed.

### Statistical analysis

Graphic and statistical analyses were performed using Microsoft Excel and Prism software. Western blot data and axonal damage was quantified using NIH ImageJ software. Data are presented as mean ± standard error of the mean (SEM). Statistical significance was calculated using Student’s *t* test or two-way ANOVA plus Bonferroni post hoc test as indicated in figure legends. *p < 0.05, **p < 0.01, ***p < 0.001, ns = not significant.

## Results

### Epo mRNA is elevated in EAE spinal cord

**Fig. 1:**
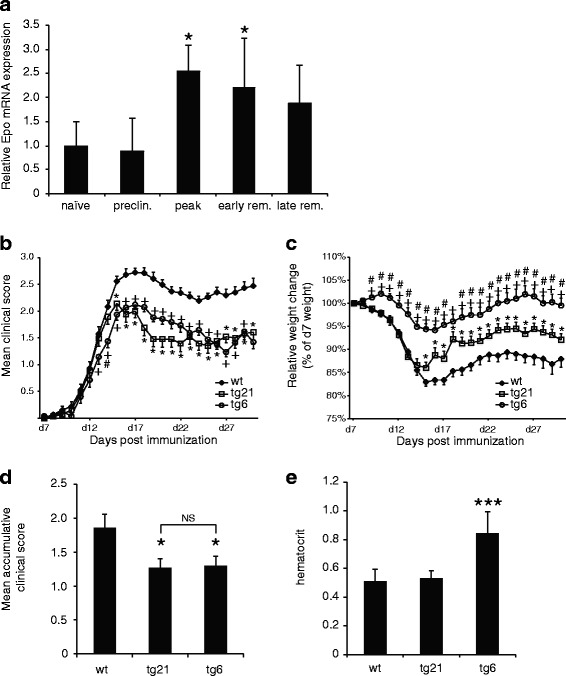
EAE disease progression and weight loss in wt, tg6 (rhEpo expression in CNS and periphery), and tg21 mice (rhEpo expression in CNS only). **a** Quantitative RT-PCR analysis of Epo mRNA expression in spinal cord at different EAE stages in wt mice. *n* = 3, Student’s *t* tests versus naïve. **b** EAE progression monitored in mice immunized with MOG using a standard method that scores the degree of paralysis on a 1 to 5 scale. **c** Weight fluctuations of the same group of mice during disease. Start weight was set to 100%. In all strains, the onset of weight loss correlates well with the onset of disability whereas the partial recovery from disability coincides with a gain in weight. **d** Mean accumulative clinical score. **e** Hematocrit measurements at the end of experiment. Combined results of six independent experiments are shown. *n* = 39 tg21, 25 tg6, and 75 wt (**b**, **c**) ANOVA, *significant difference wt versus tg21 (*p* < 0.05 or more), + significant difference wt versus tg6 (*p* < 0.05 or more), # significant difference tg21 versus tg6 (*p* < 0.05 or more); (**d**, **e**) *t* test versus wt *p < 0.05, ***p < 0.001

In the inflamed CNS, endogenous Epo has been found to be elevated possibly in direct response to insult [18, 22, 25, 52]. This rise in Epo expression is speculated to have neuroprotective properties and may have a palliating effect on neuroinflammation. We also observed Epo induction during MOG-induced EAE progression in C57Bl6, a model system of severe neuroinflammation (Fig. 1a). Examination of Epo expression in the spinal cord by quantitative RT-PCR showed that at peak disease on day 17 post-immunization (p.i.) Epo mRNA levels are significantly elevated (approximately 2.5-fold). In the early remission phase (d24 p.i.), Epo mRNA is still elevated but then declines at late-stage EAE (d30 p.i.). At this late stage, Epo mRNA levels are not significantly different from those of naïve or immunized mice before any clinical signs appear (preclin. d7 p.i.; Fig. 1a).

### Moderate rhEpo expression in the CNS is sufficient to provide amelioration from EAE

Several studies have shown that, in general, supplementing mice or rats with exogenous Epo reduces the overall severity of EAE [25, 26, 30–34, 36, 37]. It is currently unclear, however, if this is due to modulation of the peripheral immune response, alleviation of neuroinflammation, or the result of neuroprotection solely in the CNS. Therefore, we subjected tg6 mice that express rhEpo at high levels in both the periphery and the CNS, and tg21 mice in which rhEpo expression is moderate and limited to the CNS [43, 46], as well as littermate control wt mice to EAE and monitored disease progression (Fig. 1b) and weight loss (Fig. 1c). Notably, tg6 mice suffer from significantly elevated hematocrit as a consequence of high Epo concentrations, while hematocrit of tg21 mice is at wt levels (Fig. 1e). After immunization with MOG, onset of EAE occurred around the same day in all strains (Fig. 1b, d8 p.i.) and the initial increase in EAE severity progressed at a comparable rate. In tg21 mice, EAE peaked at d15 p.i. with an average score of 2.12, whereas in tg6 mice the disease peaked at d17 p.i. with an almost identical score of 2.14. In contrast, EAE severity in wt animals rose to an average score of 2.72 at d17 p.i. Average scores differed significantly between tg6 and wt mice from dpi 13 onwards and between tg21 and wt mice from d15 p.i. onwards (*p* < 0.05). Disease scores between tg6 and tg21 showed a significant difference only on d14 p.i. (*p* < 0.05). These data and the significantly higher mean accumulative clinical score in wt mice (Fig. 1d), reveal a comparable amelioration of EAE in tg6 and tg21 mice. Weight loss monitored over the course of disease correlated well with disease progression in all strains (Fig. 1c). The lowest average weight was recorded in all strains on d15 p.i. Control mice lost 17% of their initial weight recorded on d7 p.i. and tg21 mice lost a comparable 14%. Remarkably, tg6 mice displayed only minor weight loss (6%). Two-way ANOVA analysis shows that wt mice lost significantly more weight than both transgenic strains, although tg21 mice did lose significantly more weight than tg6 mice.

Since disruption of the blood-brain barrier can be anticipated in EAE, we performed several control experiments measuring rhEpo protein levels in serum and CNS lysates (Additional file 1: Figure S1). Importantly, in tg21 mice, rhEpo protein remains confined to the CNS during EAE, being detected in brain and spinal cord lysates at constant concentrations (Additional file 1: Figure S1C and B, respectively) but not in serum (Additional file 1: Figure S1A), thus excluding peripheral effects of rhEpo on EAE progression due to blood-brain barrier (BBB) leakage. Curiously, in tg6 mice, reduced rhEpo serum levels were observed at peak disease (Additional file 1: Figure S1A) although CNS levels stayed constant (Additional file 1: Figure S1B and C). All samples emphasized the six- to eightfold higher rhEpo expression levels in tg6 mice compared to tg21 mice (Additional file 1: Figure 1).

Taken together, these data strongly indicate that a modest increase in Epo concentrations restricted to the CNS, as found in tg21 mice, is sufficient to confer the maximum alleviation from EAE. Higher Epo levels in the periphery and/or CNS, as found in tg6 mice, do not augment alleviation.

### Cytokine expression in the CNS at peak EAE is altered in tg6 but not in tg21 mice

**Fig. 2:**
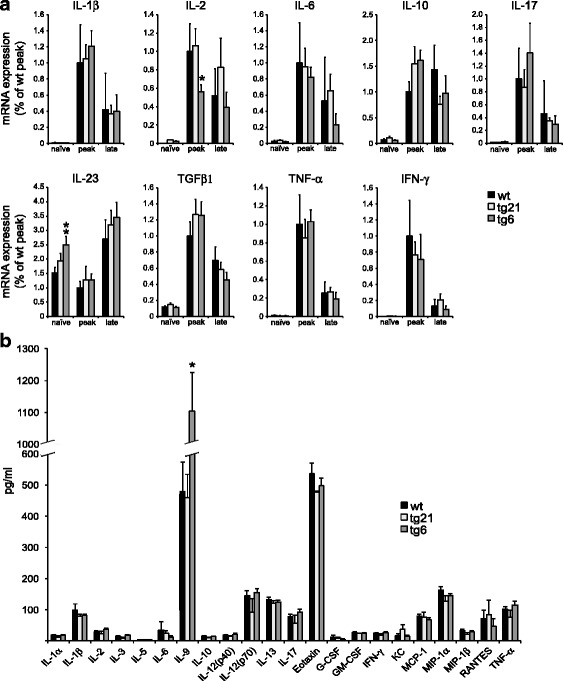
Cytokine and chemokine expression profiles during EAE in wt, tg6, and tg21 mice. **a** Quantitative RT-PCR expression analysis of a broad panel of cytokine mRNAs in spinal cord at different EAE stages in the various rhEpo-expressing mouse lines. *n* = 5 tg21, 5 tg6 and 10 wt, *t* test versus peak wt **p < 0.001. **b** Multiplex analysis of cytokine and chemokine protein amounts in spinal cord lysates from wt, tg21 and tg6 mice at late-stage EAE. *n* = 3, *t* test versus wt *p < 0.05

During both MS and EAE, the CNS is subjected to a steep increase in the concentration of disease-mediating pro-inflammatory cytokines followed by a wave of disease alleviating anti-inflammatory cytokines at later stages. Since Epo could potentially trigger changes in the CNS expression of cytokines at all EAE stages, we analyzed cytokine mRNA expression in the different strains in naïve mice and EAE-diseased mice at peak and late-stage disease (Fig. 2a). Real-time quantitative PCR showed that cytokine expression in the CNS is comparable in all strains at all EAE stages with the notable exception of IL-2, which is found at lower concentrations in tg6 animals at peak EAE, and IL-23 which is elevated in naïve tg6 animals. In addition, we analyzed CNS protein expression of a broader panel of cytokines and chemokines at late-stage EAE by multiplex (Fig. 2b). With the notable exception of IL-9 protein that was found at threefold higher concentrations in tg6 mice, no significant differences were detected in any of the other cytokines or chemokines. Notably, the comparable cytokine and chemokine profiles in wt and tg21 mice suggests that, despite the differences observed in tg6 mice, Epo does not ameliorate EAE by modulating cytokine and/or chemokine expression.

### Leukocyte populations are differentially affected in tg6 versus tg21 mice during EAE

**Fig. 3:**
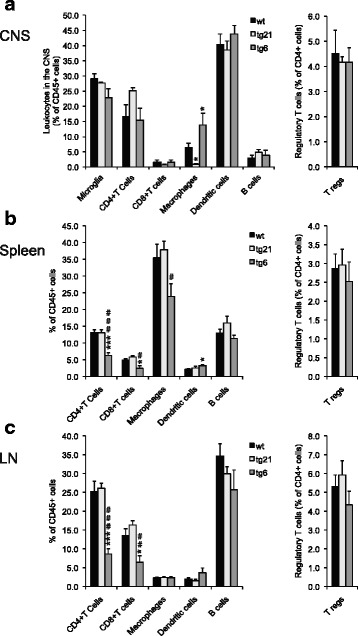
Leukocyte populations at peak EAE in the CNS, spleen, and inguinal lymph nodes of wt, tg6, and tg21 mice. Flow cytometric analysis at peak EAE of **a** CNS-infiltrating leukocytes, **b** leukocytes in the spleen, and **c** leukocytes in the inguinal lymph nodes (LN). For gating strategy and definition of leukocyte populations see the “Methods” section. *n* = 6 tg21, 6 tg6, and 12 wt. For all statistical analysis, ANOVA versus wt. *Significance between wt and tg and # between tg21 and tg6

Since the expression of rhEpo may modulate the cellular inflammatory response, we conducted an in-depth analysis of different leukocyte subsets over the course of EAE in the CNS, spleen and inguinal lymph nodes (LN) by flow cytometry (Fig. 3 and Additional file 2: Table S1). Importantly, in the CNS at peak EAE, no significant differences were detected between the different mice strains in any of the leukocyte populations investigated with the notable exception of the macrophage population, which was significantly decreased in tg21 mice and, paradoxically, increased in tg6 mice as compared to wt mice (Fig. 3a). In the tg6, periphery differences were also observed; in the LN and spleen, both the CD4 and CD8 T cell populations were reduced (Fig. 3b, c). In addition, the macrophage population was significantly smaller in the spleen and the dendritic population marginally upregulated (Fig. 3b).

At late-stage EAE, CD4 T cell numbers were reduced in the spleen and LN of tg6 mice. In addition, both CD8 T cell and B cell numbers were reduced in the spleen. In contrast, dendritic cell and macrophage populations were increased in LN of tg6 mice at late-stage EAE (Additional file 2: Table S1). In the CNS at late-stage EAE, except for an increase in CD8 T cell and macrophage numbers, the leukocyte populations in tg21 mice were very comparable to those in wt mice. Crucially, in tg21 mice no differences were observed in any of the leukocyte populations in the periphery at any EAE stage as compared to wt (Additional file 2: Table S1).

In summary, the flow cytometry data shows that the cellular immune response in tg6 mice differs from the response in wt mice primarily in the periphery. In contrast, leukocyte populations in tg21 mice do not differ from wt mice in any of the peripheral organs. Finally, the changes observed in macrophage and CD8 population in the CNS of tg21 mice are not detected in tg6 mice.

### rhEpo expression does not prevent myelin damage or promote remyelination in EAE

**Fig. 4:**
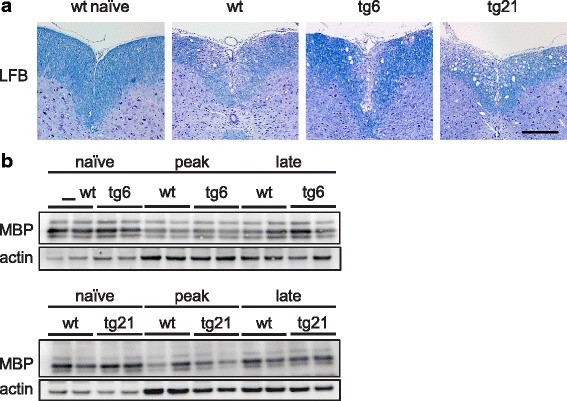
Myelin loss and remyelination in wt, tg6, and tg21 mice during EAE. **a** Luxol fast blue myelin staining of lumbar and thoracic spinal cord from naïve wt mice and wt, tg21, and tg6 mice at late-stage EAE. Scale bar = 200 μm. **b** Representative Western blot analysis of CNS lysates from 2 naïve and 2 EAE diseased tg21, tg6 and wt mice for myelin basic protein (MBP). Reduced intensity of MBP bands at peak EAE indicates myelin loss, whereas the increase in intensity at late-stage EAE indicates remyelination

Reduced EAE severity in tg6 and tg21 mice could be explained by an amending effect of rhEpo expression on myelin damage, remyelination, or astrocyte activation. Therefore, in a first approach, we performed Western blot analysis of spinal cord lysates harvested from naïve mice and animals at peak and late-stage EAE. In all mice, myelin basic protein (MBP) expression, representing a proxy of myelin integrity, was diminished at peak EAE followed by some recuperation at late stages (Fig. 4b). At both peak and late EAE, the MBP levels in transgenic and wt spinal cord lysates were comparable, suggesting a similar scale of myelin loss and remyelination in both strains. In good agreement, luxol fast blue histology of lumbar and thoracic spinal cord harvested from mice at late-stage EAE also revealed a similar degree of myelin damage in wt, tg6, and tg21 mice (Fig. 4a). Thus, rhEpo expression does not significantly prevent myelin damage or promote remyelination during EAE.

### rhEpo expression limits neuronal loss in EAE

Neuroprotective properties have been attributed to exogenous Epo administration in several in vitro and in vivo experimental models of neurodegeneration including various EAE models [20, 30, 53, 54]. To investigate if endogenous expression of rhEpo in the CNS confers neuroprotection in EAE, we first performed Western blot analysis of brain lysates harvested from naïve mice and animals at peak and late-stage EAE. In all mice, changes in pan-neurofilament (NF), phosphorylated (smi35), and non-phosphorylated neurofilament (smi32) expression, representing a proxy of neuronal integrity, were significantly diminished at peak EAE followed by some recuperation at late stages (Additional file 3: Figure S2). Notably, expression levels of these neurofilament species were comparable between transgenic and wt mice at all EAE stages.

**Fig. 5:**
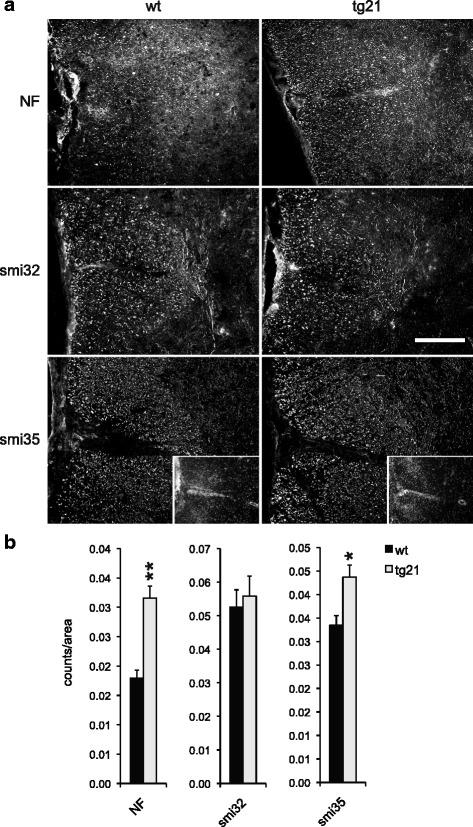
Neuronal loss in the spinal cords of wt, tg6, and tg21 mice at late-stage EAE. **a** Immunohistochemical analysis of lumbar and thoracic spinal cord sections for neurofilament (NF), non-phosphorylated neurofilament H (smi32), and phosphorylated neurofilament H and M (smi35). Inserts in smi35 panels show DAPI co-stain revealing leukocyte infiltration into the spinal cord. Scale bar = 200 μm (**b**) Quantitative analysis of immunohistochemistry shown in **a**. NF: wt *n* = 4, tg21 *n* = 3; smi32: wt *n* = 3, tg21 *n* = 3; smi35: wt *n* = 4, tg21 *n* = 4. Four sections per animal from lumbar and thoracic spinal cord were analyzed. *t* test versus wt *p < 0.05, **p < 0.01

The EAE model we used in this study is characterized by extensive inflammation of the spinal cord rather than inflammation of the brain. Therefore, we performed a detailed immunohistochemical analysis and quantitation on the cellular level of thoracic and lumbar spinal cord of wt and tg21 mice (Fig. 5). Although pan-neurofilament staining of lumbar and thoracic spinal cords harvested from mice at late-stage EAE and subsequent quantitative image analysis shows loss of dorsal funicular axons in both wt and tg21 mice, axonal loss was significantly more extensive in wt animals (NF, Fig. 5a, b). We also used anti-smi35 antibody that reacts primarily with phosphorylated neurofilament H and M and stains thick and thin axons and, albeit faintly, cell bodies. Using this marker, comparable results were obtained again revealing more extensive loss of dorsal funicular axons in wt mice (smi35, Fig. 5a, b). Importantly, although neuronal loss in wt mice was more extensive, co-staining with DAPI indicated a similar degree of leukocyte infiltration in wt and tg21 mice (Inserts in smi35 pictures of Fig. 5a). Smi32 immunohistochemistry was used to assay the extent of axonal damage (smi32, Fig. 5a). In contrast to smi35, smi32 stains mainly damaged neurons by reacting with a non-phosphorylated epitope in neurofilament H, which is masked by phosphorylation in healthy neurons. Surprisingly, quantitative analysis revealed a comparable number of damaged axons in wt and tg21 mice (smi32 Fig. 5a, b).

In summary, damaged axons were detected at late-stage EAE in the spinal cord of both wt and tg21 mice; however, axonal loss was significantly more prominent in wt animals.

## Discussion

To date, several rodent studies have presented evidence for a beneficial effect of exogenous Epo treatment in EAE [25–27, 30–38]. However, the underlying mechanisms by which its palliating effects are mediated remain a matter of debate. Unfortunately, several human trials have produced mixed results, most likely due to the small and heterogeneous patient groups enrolled [40–42]. Clearly better mechanistic insight into the beneficial effect of Epo treatment in EAE may help determine the best approach to treatment of MS patients with Epo (or its derivatives). Using two transgenic mouse models that either overexpress Epo systemically or limited to the CNS, we show for the first time that prevention of neuronal loss in the spinal cord appears to be the primary advantage of Epo treatment in EAE, rather than immunomodulation.

We and others [52] have observed significantly elevated Epo mRNA levels in the spinal cord at peak disease, when leukocyte infiltration into the CNS is most prominent and pro-inflammatory cytokine levels are high. These levels slowly decrease at late-stage EAE coinciding with a decline in CNS-infiltrating leukocytes and a shift to anti-inflammatory cytokine expression. This timing strongly suggests that neuroinflammation triggers these events and highlights an inherent protective response of the CNS to injury. This is further underlined by the fact that exogenously applied Epo [25, 26, 30–34, 36, 37] and overexpression of Epo as in this study clearly benefits outcome by reducing disease severity. Clearly, Epo-mediated suppression of the inflammatory response in the periphery and/or CNS, altering the cellular immune response and cytokine/chemokine expression, could explain its beneficial effects. However, a positive effect of Epo on neuroprotection/regeneration, myelin protection, remyelination, or microglial responses would explain the benefits equally well. Although global rhEpo expression in tg6 mice modulates the peripheral immune response, this effect does not appear to contribute significantly to improved EAE outcome because modest rhEpo expression restricted to the CNS (tg21 mice) results in comparable disease alleviation without altering the peripheral immune response. Direct peripheral effects of rhEpo in tg21 mice can be excluded as increases in hematocrit, rhEpo in the blood, or changes in the immune compartment were not detected under any circumstances indicating that possible leakage of rhEpo from the CNS due to blood-brain barrier breakdown did not occur. As the EAE disease course was comparable in both lines, a disease improvement mainly due to Epo-mediated peripheral immunomodulation can be clearly excluded.

Of all the CNS leukocyte populations assayed, apart from an increase in CD8 T cells in tg21 mice at late-stage EAE, only the macrophage population was affected in transgenic animals compared to wt animals. However, where tg21 mice showed a decrease in macrophages at peak and an increase at late EAE as compared to wt, we found the opposite result in tg6 animals with an increase at peak and no significant difference at late-stage EAE. Taken together, these results suggest that potential Epo-triggered alterations in leukocyte population within the CNS are unlikely to explain the beneficial effects of Epo on EAE progression.

In summary, although various reports suggest Epo has a direct effect on the immune response resulting in alleviation of disease, our study does not reproduce the details of these findings. For example, Li et al. observed a reduction of spinal cord major histocompatibility complex (MHC) class II expression in Epo-treated animals that they correlated with reduced microglia activation [33]. However, in all our mice, a comparable number of microglia in the CNS at all EAE stages was seen arguing against a major effect of Epo on microglia activation. Similarly, we did not detect any changes in regulatory T cells in our mice, in contrast to a report showing Epo can promote expansion of regulatory T cell populations [32]. It is notable however that all these previous works used exogenous short-duration Epo therapies whereas this study used a constitutively overexpression of Epo.

In addition to changes in the cellular immune response, several observations in tg6 mice on the cytokine/chemokine level are notable. For example, IL-23 mRNA is significantly elevated in the CNS of naïve tg6 mice indicating that constitutively high CNS Epo levels trigger IL-23 expression. How Epo triggers IL-23 expression, and in which cells, is at present unclear. Indeed, it is remarkable that the elevated IL-23 mRNA amounts in tg6 mice have no additional exacerbating effect since IL-23 is an essential cytokine in EAE development promoting the expansion of pathogenic myelin-specific IL-17 T cells (reviewed in [55–57]). Similarly, although significantly lower IL-2 mRNA levels were found in the CNS of tg6 mice, and thus lower CNS-infiltrating T cell numbers were anticipated, the cellular immune response in the CNS proved comparable between strains. These conundrums suggest the possibility that other cytokines such as IL-9 or IL-15 compensate for the reduced IL-2 amounts. Indeed, multiplex showed IL-9 levels were elevated in tg6 mice. It is still debated whether IL-9 contributes positively [58] or negatively [59, 60] to the pathogenesis of EAE; however, our data suggests that high IL-9 levels do not exacerbate disease score or increase the number of leukocytes in the CNS. Overall, it appears that Epo-mediated immunomodulation does not significantly impact disease outcome.

Our data indicates that Epo mediates its positive effects in EAE predominantly by neuroprotection in the spinal cord, particularly by preventing neuronal loss but not necessarily inhibiting neuronal damage. Indeed Epo has a well-established neuroprotective role in a number of disease paradigms including neonatal or adult rodent focal brain ischemia, brain trauma, and spinal cord injury (reviewed by [22]). Generally, Epo protects neuronal cells by regulating the balance between proapoptotic and antiapoptotic pathways. In agreement, we noted significantly induced levels of the proapoptotic protein Bax in wt animals were suppressed in the transgenic lines (data not shown) showing a tipping of the balance towards protection in the Epo-overexpressing animals. However, a whole array of protective mechanisms is likely to be activated as suggested by a number of reports [20, 53, 54, 61–63]. As we cannot exclude the possibility that permanent overexpression of EPO in the brain might lead to physiological adaptive changes that are undetected in our study, further investigation is needed to fully understand Epo’s neuroprotective mechanism of action during EAE progression.

Although putative regeneration-enhancing effects are less well studied in vivo, a few reports have implicated Epo may promote the production of neuronal and oligodendrocyte progenitors [25, 64, 65] and improve recovery of neurological function [66]. Neurogenesis was not investigated in this study and so cannot be excluded, but our data surprisingly shows that myelin damage at late-stage EAE is not significantly different between wt and transgenic mice. This suggests that Epo does not ameliorate EAE through prevention of myelin damage, or augmentation of remyelination.

Interestingly, our data and that of others [52] indicate that the CNS inherently responds to neuroinflammation with an increase in locally produced Epo, which possibly serves as a neuroprotective mechanism. The data presented herein further underlines that a relatively modest increase in CNS Epo, in addition to the endogenous increase in response to neuroinflammation, could significantly enhance this neuroprotective effect to obtain maximum palliation. Several groups in academia and the pharmaceutical industry have explored CNS-specific Epo receptor agonists that have no, or low, efficacy for peripheral Epo receptors to avoid inducing elevated hematocrit levels [16, 67, 68]. Undoubtedly, such agents would provide invaluable benefit for the treatment of CNS disorders. An alternative approach could be to locally increase Epo levels in the CNS. For example, astrocytes are likely to be largely responsible for the increase in CNS Epo levels in response to neuroinflammation. Identification of a pathway that triggers astrocytic Epo production could be used to develop therapeutic approaches that increase CNS Epo without increasing Epo levels in the periphery. Such an approach would avoid side effects on the peripheral immune system and circumvent detrimental increases in hematocrit. Notably, the lack of a phenotype in unchallenged tg21 mice suggests that modest constitutive overexpression of Epo in the CNS would be well tolerated.

## Conclusions

This study demonstrates that the primary beneficial effect of Epo on EAE disease progression resides in the CNS. Despite the extensively investigated effects of Epo on the immune system, our data indicates that the neuroprotective effects of Epo treatment in EAE are sufficient to provide maximum alleviation. Notably, only a moderate increase of Epo levels in the CNS was required for a positive outcome. With these insights, this study advocates that use of Epo (and neuroprotective derivatives of Epo) as a future therapeutic for neurological disease should not be dismissed.

## Additional files

Additional file 1: Figure S1. Recombinant human Epo levels in serum and CNS of naïve and of EAE-diseased tg6 and tg21 mice. rhEpo levels were measured by ELISA in (**A**) serum, (**B**) spinal cord and (**C**) brain lysates of naïve and EAE-diseased tg6 and tg21 mice at peak and at late stage EAE. n ≥ 4, *t* test versus strain naïve *p < 0.05, **p < 0.01. (PDF 332 kb)

Additional file 2: Table S1. Flow cytometric analysis at peak and late stage EAE of leukocytes populations in the CNS, spleen and the inguinal lymph nodes (LN). For gating strategy and definition of leukocyte populations see Materials and Methods. Student’s *t* test *p < 0.05, **p < 0.01, *p* < 0.001, ns = not significant. (PDF 63 kb)

Additional file 3: Figure S2. Western blot analysis of pan-, non-phosphorylated, and phosphorylated neurofilament protein levels in the CNS of wt, tg6 and tg21 mice during EAE. Representative Western blot analysis of CNS lysates from 2 naïve and 2 EAE-diseased (**A**) wt and tg6 mice, and (**B**) wt and tg21 mice, for neurofilament (NF), non-phosphorylated neurofilament H (smi32) and phosphorylated neurofilament H and M (smi35). Graphs show data from *n* = 4, t-test. (PDF 488 kb)
