# Supplementary figures and images for: Erythropoietin reduces experimental autoimmune encephalomyelitis severity via neuroprotective mechanisms
Source: J Neuroinflammation. 2017 Oct 13;14:202. doi: 10.1186/s12974-017-0976-5 (PMC5640948; doi:10.1186/s12974-017-0976-5)

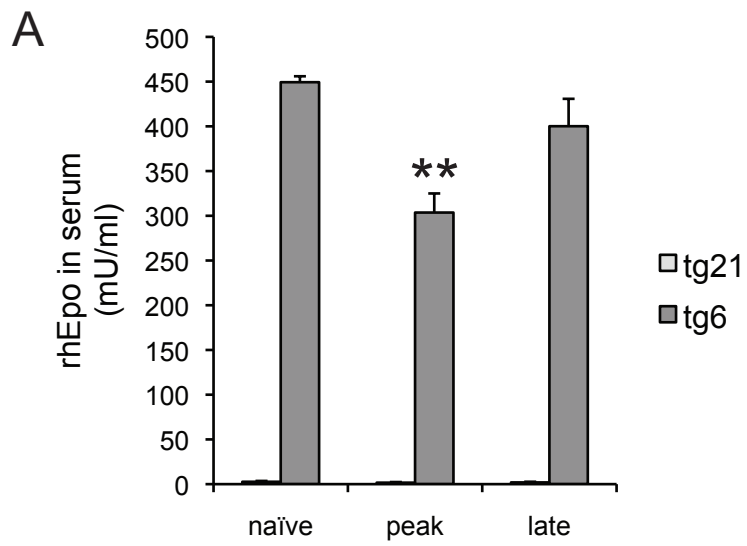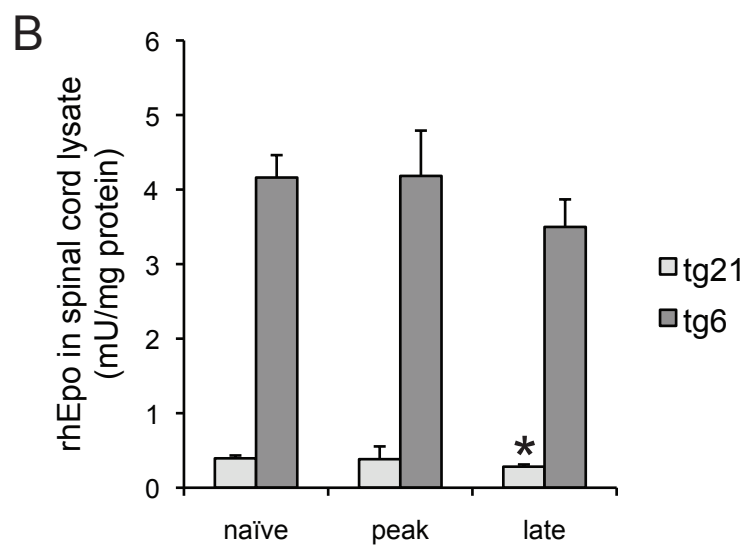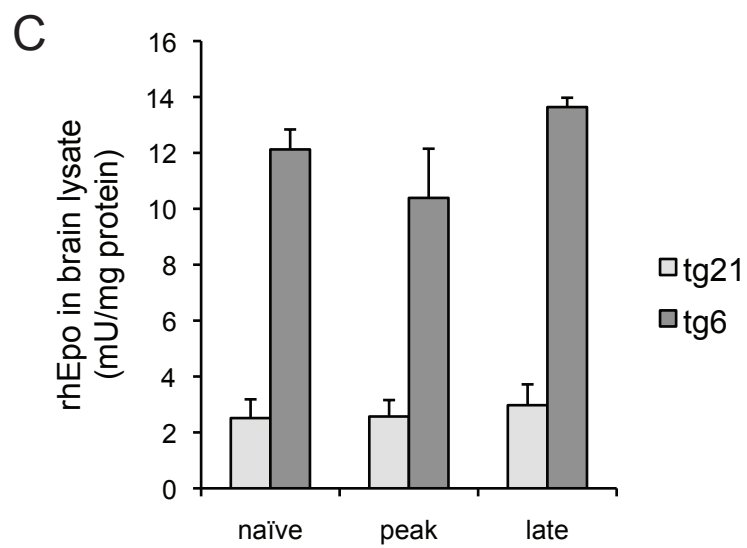

Supplement: Supplementary file 1 — Recombinant human Epo levels in serum and CNS of naïve and of EAE-diseased tg6 and tg21 mice. rhEpo levels were measured by ELISA in (A) serum, (B) spinal cord and (C) brain lysates of naïve and EAE-diseased tg6 and tg21 mice at peak and at late stage EAE. n ≥ 4, t test versus strain naïve *p < 0.05, **p < 0.01. (PDF 332 kb) [file 12974_2017_976_MOESM1_ESM.pdf]

**A**

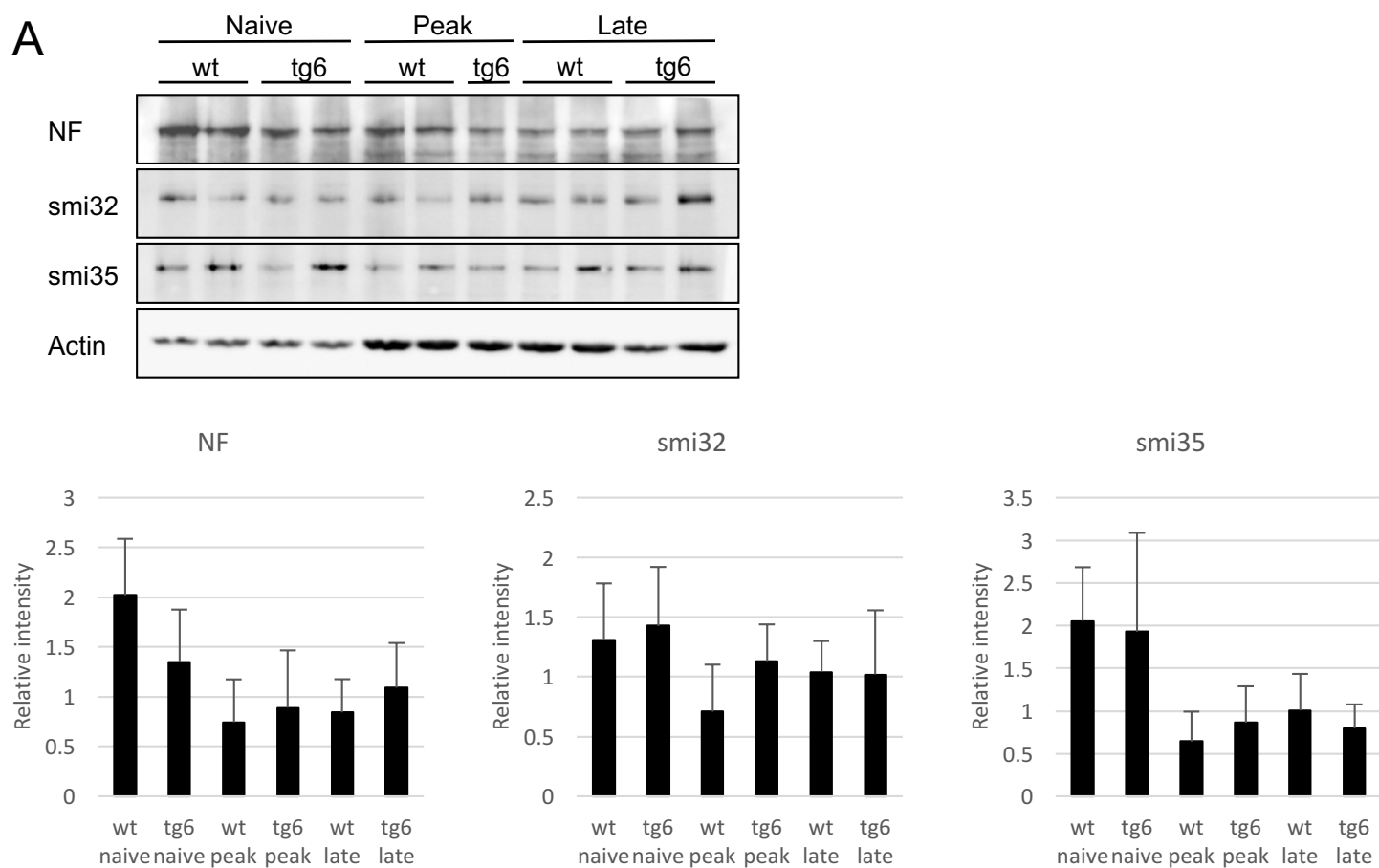

**B**

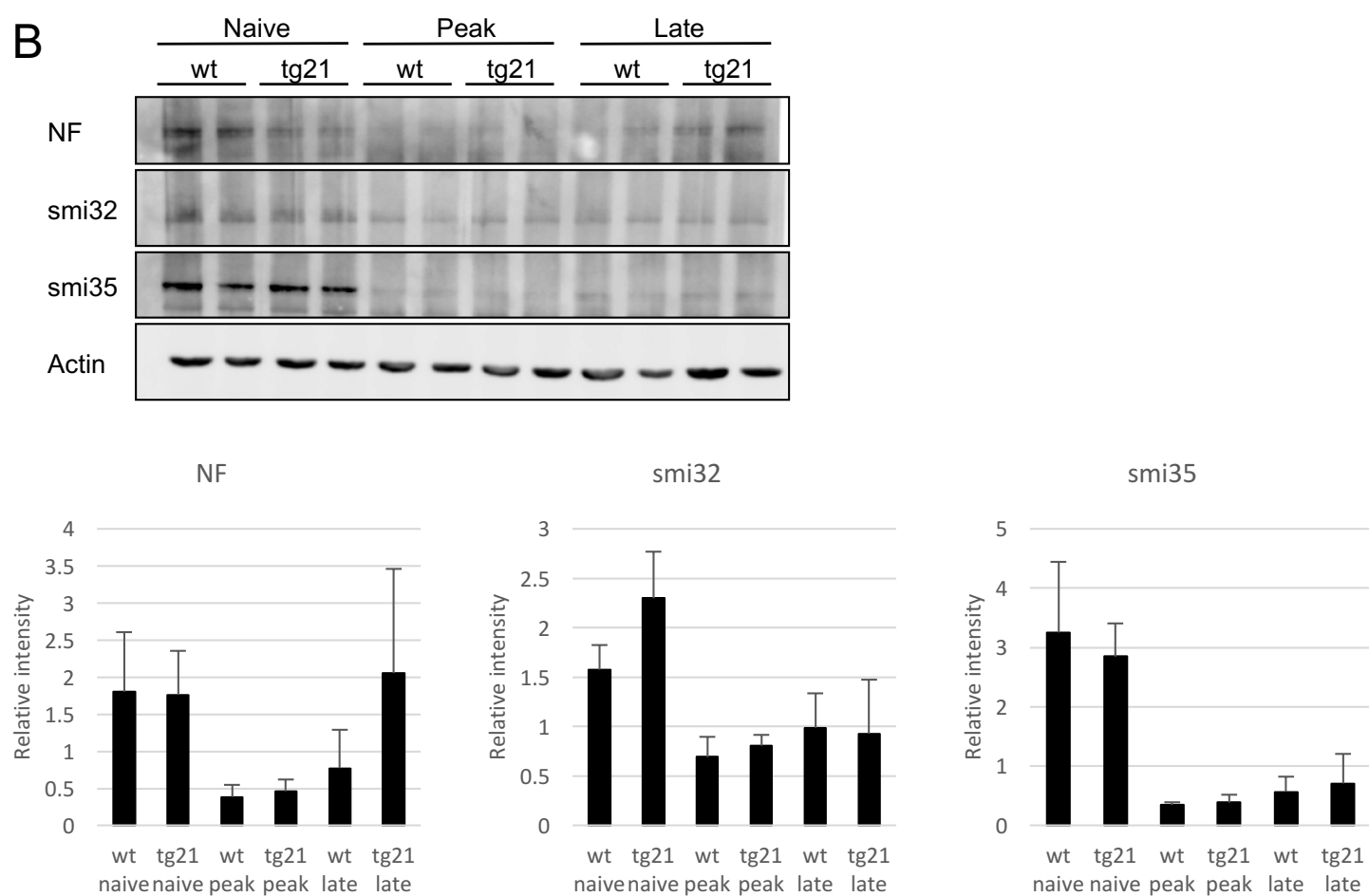

Supplement: Supplementary file 3 — Western blot analysis of pan-, non-phosphorylated, and phosphorylated neurofilament protein levels in the CNS of wt, tg6 and tg21 mice during EAE. Representative Western blot analysis of CNS lysates from 2 naïve and 2 EAE-diseased (A) wt and tg6 mice, and (B) wt and tg21 mice, for neurofilament (NF), non-phosphorylated neurofilament H (smi32) and phosphorylated neurofilament H and M (smi35). Graphs show data from n = 4, t-test. (PDF 488 kb) [file 12974_2017_976_MOESM3_ESM.pdf]
