# Supplementary material for: Erythropoietin reduces experimental autoimmune encephalomyelitis severity via neuroprotective mechanisms
Source: J Neuroinflammation. 2017 Oct 13;14:202. doi: 10.1186/s12974-017-0976-5 (PMC5640948; doi:10.1186/s12974-017-0976-5)
Supplement: Supplementary file 2 — Flow cytometric analysis at peak and late stage EAE of leukocytes populations in the CNS, spleen and the inguinal lymph nodes (LN). For gating strategy and definition of leukocyte populations see Materials and Methods. Student’s t test *p < 0.05, **p < 0.01, p < 0.001, ns = not significant. (PDF 63 kb) [file 12974_2017_976_MOESM2_ESM.pdf]

Peak EAE	Population (% of CD45)	wt Mean	wt SEM	wt N	tg21 Mean	tg21 SEM	tg21 N	tg6 Mean	tg6 SEM	tg6 N	student's ttest wt vs tg21	student's ttest wt vs tg6	student's ttest tg21 vs tg6
CNS	Microglia	28.97	1.69	9	27.72	0.27	3	22.93	3.01	6	ns	ns	ns
	CD4+T Cells	16.49	3.92	9	25.10	1.01	3	15.48	3.88	6	ns	ns	ns
	CD8+T cells	1.54	0.79	9	0.76	0.25	3	1.61	0.68	6	ns	ns	ns
	Macrophages	6.52	1.21	9	0.95	0.14	3	13.91	3.92	6	*	*	ns
	Dendritic cells	40.40	3.49	9	38.55	3.03	3	43.89	2.73	6	ns	ns	ns
	B cells	2.92	0.93	9	4.78	1.01	3	4.01	1.42	6	ns	ns	ns
	T regs (% of CD4)	4.51	0.94	9	4.16	0.22	3	4.17	0.58	6	ns	ns	ns
Spleen	CD4+T Cells	13.11	0.87	12	13.08	0.89	6	6.27	0.82	6	ns	***	***
	CD8+T cells	4.84	0.51	12	5.85	0.38	6	2.42	0.76	6	ns	*	*
	Macrophages	35.44	4.08	12	37.88	2.56	6	23.88	3.86	6	ns	ns	*
	Dendritic cells	2.14	0.27	12	2.55	0.53	6	3.25	0.36	6	ns	*	ns
	B cells	12.97	1.14	12	15.98	2.01	6	11.35	0.95	6	ns	ns	ns
	T regs (% of CD4)	2.86	0.39	12	2.96	0.42	6	2.52	0.52	6	ns	ns	ns
LN	CD4+T Cells	25.16	2.78	12	26.12	1.32	6	8.64	1.36	6	ns	***	***
	CD8+T cells	13.49	1.87	12	16.37	1.10	6	6.47	1.73	6	ns	*	**
	Macrophages	2.30	0.18	12	2.41	0.28	6	2.36	0.34	6	ns	ns	ns
	Dendritic cells	1.96	0.38	12	1.52	0.38	6	3.66	1.26	6	ns	ns	ns
	B cells	34.67	3.28	12	29.93	1.91	6	25.72	5.22	6	ns	ns	ns
	T regs (% of CD4)	5.32	0.61	12	5.93	0.76	6	4.34	0.73	6	ns	ns	ns

Late EAE	Population (% of CD45)	wt Mean	wt SEM	wt N	tg21 Mean	tg21 SEM	tg21 N	tg6 Mean	tg6 SEM	tg6 N	student's ttest wt vs tg21	student's ttest wt vs tg6	student's ttest tg21 vs tg6
CNS	Microglia	21.82	2.09	12	27.17	2.89	6	18.72	0.70	6	ns	ns	*
	CD4+T Cells	15.41	1.56	12	17.52	1.05	6	14.78	1.45	6	ns	ns	ns
	CD8+T cells	0.92	0.25	12	1.89	0.29	6	0.54	0.28	6	*	ns	*
	Macrophages	2.20	0.37	12	5.23	1.28	6	1.76	0.53	6	*	ns	*
	Dendritic cells	57.80	3.73	12	47.42	4.36	6	59.92	7.42	6	ns	ns	ns
	B cells	3.88	0.65	12	4.62	0.54	6	7.00	2.09	6	ns	ns	ns
	T regs (% of CD4)	11.59	2.85	9	10.70	2.96	6	10.35	0.93	3	ns	ns	ns
Spleen	CD4+T Cells	13.91	1.33	12	16.18	2.78	6	4.55	0.32	5	ns	***	*
	CD8+T cells	7.10	1.01	12	8.86	1.92	6	3.11	0.99	6	ns	*	*
	Macrophages	14.24	3.15	12	10.36	4.50	6	19.97	2.52	6	ns	ns	ns
	Dendritic cells	3.20	0.61	12	1.66	0.45	6	4.35	0.39	6	ns	ns	**
	B cells	23.24	2.27	12	29.57	2.47	6	10.36	2.36	6	ns	*	***
	T regs (% of CD4)	2.52	0.49	12	4.17	0.60	6	1.83	0.56	6	ns	ns	*
LN	CD4+T Cells	20.10	0.74	10	21.92	1.65	6	15.00	1.31	4	ns	*	*
	CD8+T cells	16.46	1.30	10	15.67	2.52	6	11.86	2.00	4	ns	ns	ns
	Macrophages	1.85	0.22	10	2.01	0.21	6	2.90	0.51	4	ns	*	ns
	Dendritic cells	2.13	0.24	10	1.95	0.50	6	3.61	0.78	4	ns	*	ns
	B cells	32.06	1.47	10	31.52	2.30	6	37.65	4.63	4	ns	ns	ns
	T regs (% of CD4)	5.67	0.66	10	4.94	0.17	6	6.13	1.07	4	ns	ns	ns
